# Enhancing Chicken Meat Quality with User-Friendly Decontamination Wipes

**DOI:** 10.3390/foods14193391

**Published:** 2025-09-30

**Authors:** Suman Talukder, Arup Ratan Sen, Immanuel Prince Devadason, Ashim Kumar Biswas, Murthy Suman Kumar, Himani Dhanze, Kiran Narayan Bhilegaonkar, Hung Nguyen, Delia Grace, Ram Pratim Deka

**Affiliations:** 1 ICAR-Indian Veterinary Research Institute, Bareilly 243122, India; senarup68@gmail.com (A.R.S.); princenadar@yahoo.co.in (I.P.D.); biswaslpt@gmail.com (A.K.B.); sumanvph@gmail.com (M.S.K.); hdhanze@yahoo.co.in (H.D.); kiranvph@rediffmail.com (K.N.B.); 2 International Livestock Research Institute, P.O. Box 30709, Nairobi 00100, Kenya; h.nguyen@cgiar.org (H.N.); d.randolph@cgiar.org (D.G.); 3 International Livestock Research Institute, National Agricultural Science Complex, Pusa, New Delhi 110012, India

**Keywords:** dressed chicken, retail chicken meat, microbial contamination, decontamination wipe, improved quality

## Abstract

The unhygienic practices in retail poultry meat shops adversely affect chicken meat quality and shelf life. To address this issue, a meat-surface-decontaminating wipe was developed. Deionized water, jamun leaf (*Syzygium cumini*) extracts, and other generally recognized as safe ingredients were used to prepare a decontamination solution. A sterile non-woven cloth soaked in the solution was applied over the meat surface as a decontamination wipe. Treated and untreated meat samples were stored at 4 ± 1 °C under aerobic packaging conditions, and various meat quality parameters were evaluated at every 1-day interval until the onset of spoilage. It was observed that the wipe could significantly reduce 2.31 log microbial loads (*p* = 0.00005, CI-95%, 1.85, 2.77) over the meat surface. With the increasing storage intervals, the meat quality parameters changed significantly (*p* < 0.05), and the treated chicken samples spoiled on day 6, whereas the control spoiled on day 5. The meat spoilage was confirmed by the evaluation of quality changes in the stored meat. Additionally, the wipe could cause 1.14 (*p* = 0.00000, CI-95%, 1.01, 1.25) and 1.03 (*p* = 0.00005, CI-95%, 0.90, 1.16) log reductions of *E. coli* and *S. aureus*, respectively. Based on the results, it was concluded that the decontamination wipe could improve the meat quality and shelf life of retail chicken meat without affecting the sensory quality attributes.

## 1. Introduction

Nutritional richness, affordability, and easy availability have made chicken meat a popular animal-sourced food item across the world [1]. The quality of broiler chicken meat is the key factor influencing consumer purchasing decisions. Several factors, such as environmental conditions, transportation, handling, slaughter technique, processing, storage, and temperature maintenance in the cold chain, influence meat quality [2]. The quality achieved through scientific slaughter and hygienic carcass handling must be consistently maintained throughout the supply chain to safeguard consumer safety [3]. Raw chicken is highly prone to contamination, primarily governed by sanitation at the retail level [4]. Furthermore, post-slaughter factors, including biochemical and microbial changes in meat, are responsible for the rapid deterioration of chicken meat quality [5]. The microbe-friendly, nutrient-rich composition and the unhygienic handling practices involved in the retailing of hot-processed chicken meat make it susceptible to quality deterioration. Poultry meat and its products are commonly associated with several pathogens like *E. coli*, *S. aureus*, *Salmonella*, *Campylobacter*, and *Listeria*. However, *Yersinia enterocolitica*, *Aeromonas*, and *Clostridium perfringens* have been identified as major pathogens in poultry-related products in some instances. Additionally, *Salmonella*, *Campylobacter*, and *Listeria* have been found to be the most common agents for food-borne illnesses in the poultry meat sector [6].

In India, the majority of chicken meat consumers purchase their meat from the wet markets and local retail shops because of its perceived freshness, cheaper price, and abundant availability. However, the lack of infrastructure facilities and information among the poultry meat retailers means that they are less aware of the microbial contamination and quality control of retail chicken. A significant proportion of the retail poultry meat handlers and processors fail to adhere to standard practices for handling live poultry birds, maintaining hygiene during the slaughter of birds, or ensuring post-slaughter sanitation. Consequently, the quality and safety of the chicken meat supplied to consumers are compromised [7]. Ultimately, this may lead to foodborne illness and the spread of infection among the chicken meat consumers [8]. Therefore, to avoid unwanted consumer health impacts, a suitable meat quality management strategy is important in the chicken meat supply chain. However, the available high-end, costly, and complicated intervention techniques are not practically applicable within the present structure of the retail poultry chain in developing countries like India.

Controlling contamination on carcass surfaces has been found to be effective in significantly reducing meat-borne human infections [9]. Additionally, meat surface disinfection has been shown to be effective in reducing microbial contamination and thereby delaying its spoilage [10]. Decontamination of meat with many techniques has been studied [11], specifically for chicken meat [12]. Treating broiler meat with different chemicals could reduce the levels of *Campylobacter* and many spoilage microbes, which results in increased product shelf life [13,14]. Surface decontamination with a novel decontaminant is a pivotal factor in improving meat quality and extending shelf life. A user-friendly, economically viable, and environmentally friendly chicken meat-surface decontamination technique was emphasized to address the current issue. According to Malheiro et al. [15], there is scope for developing a novel formulation that can kill microbes without any toxic residual effects.

The application of phytochemicals is a potential biocide for developing a surface disinfectant [16]. Researchers have shown that jamun leaves (*Syzygium cumini*), which grow commonly in Southeast Asian countries, have effective antimicrobial efficacy [17]. These leaf extracts demonstrated antimicrobial activity against *E. coli* (minimum inhibitory concentration: MIC, 512 µg/mL) and *S. aureus* (MIC ≥ 1024 µg/mL) [18]. The antimicrobial activity of the jamun leaf extracts is contributed to by the polyphenols and tannins present in it [19,20]. Additionally, this leaf has also been used in human food due to its antimicrobial capacity [21,22].

In addition to bioactive leaf extracts, essential oils from different plant sources have also shown potential antimicrobial efficacy. A previous study has proven the effectiveness of thyme essential oil (TEO) as a biopreservative for meat [23]. Several substances are generally recognized as safe (GRAS), such as organic acid [24], sodium benzoate and potassium sorbate [25], nisin [26], etc., which have been recommended for food preservation. These ingredients can effectively be used for decontaminating the food surface, and for that, a convenient base is needed for their effective application. A general observation shows that the non-woven-based dry and wet wipes are effective in hygiene, personal, and household applications [27]. Therefore, in this study, an attempt was made to develop meat-surface decontamination wipes (DWs) using deionized water, jamun leaf extract, TEO, and other GRAS ingredients with a non-woven cloth as the base material for application on chicken meat surfaces. This will address the issue of meat contamination and quality deterioration due to unhygienic handling in retail chicken meat shops. Therefore, emphasis was given to developing a user-friendly, economically viable, and environmentally friendly chicken meat surface decontamination wipe.

## 2. Materials and Methods

The jamun leaves were collected from the garden on the premises of the ICAR-IVRI, Izatnagar. Fully matured green leaves were plucked and cleaned under running tap water. The essential oil was purchased from AOS^®^ Products Pvt. Ltd., Ghaziabad, India. The non-woven cloth (50% viscose and 50% polyester) was procured from Amazon^®^, New Delhi, India. Other chemicals (analytical grade) used in this study were procured from standard firms like Merck (Mumbai, India), SRL (Mumbai, India), Thermo Fisher Scientific (Mumbai, India), Hi-Media (Thane, India), S D Fine-Chem Limited (Vadodara, India), etc. The chicken meat samples were purchased from the local market in Bareilly city.

### 2.1. Selection of Ingredients and Formulation of Decontamination Solution

To develop the DW, a decontamination solution (DS) was prepared. For that, all-natural and GRAS ingredients were chosen. Due to its neutrality, easy availability, and economic nature, sterile deionized water (SDW) was chosen as the medium for the DS.

In the present study, two pathogenic microorganisms, viz., *E. coli* and *S. aureus*, were chosen to check the antimicrobial efficacy of both the plant extracts and the developed DW. These two microbes are closely associated with chicken meat and are commonly used as indicator organisms to assess the effectiveness of hygiene practices during their processing [28,29]. *S. aureus* has been recognized as the major cause of foodborne illness. Further, *E. coli* has been found to be the most frequent facultative anaerobic microbe; its presence in foods suggests fecal contamination [30].

In the preliminary trials, jamun leaf extract was prepared following the method of Biswas et al. [31] with little modification (Figure S1), and its antimicrobial activity was evaluated against the test organisms using the well diffusion assay. The agar well diffusion test was found to be a suitable method for evaluating the antimicrobial activity of plant extracts. The zone of inhibition produced by the well diffusion test against the test microorganisms defines the antimicrobial efficacy [32]. For the well diffusion test, sterile nutrient plates (Mueller Hinton Agar—MHA) were made, and after they set for 5 min, holes were created in the plates using a well borer to make 6 mm wells. An inoculum suspension of *S. aureus* (ATCC 29213) and *E. coli* (ATCC 43888) was fixed to a 0.5 McFarland standard (10^8^ CFU/mL) and then uniformly swabbed over the surface of the solidified agar. Finally, 100 μL of jamun leaf extract and 100 μL of SDW (negative control) were loaded separately into the wells, and the plates were incubated at 37 °C for 24 h [33,34]. The antibacterial efficacy of the leaf extract was assessed by measuring the diameter of the zone of inhibition in millimeters and recorded. Elfadil et al. [17] have demonstrated that the jamun leaf extract has a potent antimicrobial efficacy against both *S. aureus* and *E. coli*.

Among other ingredients in the DS formulation, organic acids, thyme essential oil (TEO), and nisin were important due to their significant antimicrobial effect. These ingredients, along with others, were chosen based on the available literature. For TEO, its antimicrobial efficacy has been harnessed previously for preserving the meat as a GRAS ingredient [35]. Various GRAS organic acids and salts have been reported to reduce the microbial population and, at the same time, improve meat quality attributes [36,37]. The incorporation levels of the selected ingredients were based on the available literature. Glacial acetic acid, one of the ingredients of DS, shows optimal effects at 1–3% when used in a meat rinse. Citric acid at a 1% concentration can effectively reduce the mean pH without compromising the sensory attributes. Sodium chloride has been found to show meat preservation effects in 2–3% concentration; in addition, sodium bicarbonate is generally used at 1–2% in meat washes. Sodium benzoate at 0.05–1% shows effective antifungal activity, whereas addition at a higher level (0.5%) can further improve its efficacy. At a 0.1–0.3% concentration, potassium sorbate shows optimum antifungal activity. Lactic acid was found to be an effective acid rinse agent at a2–5% concentration for decontaminating *E. coli* and *Salmonella*. TEO and nisin were found to be very effective against foodborne pathogens at 0.1–0.5% and 0.05–0.2% concentrations.

To prepare 4 different DS formulations, the selected ingredients were mixed with SDW in varying proportions, namely control, F-1, F-2, and F-3 (Table 1). The control formulation (C) was made only with the plant extracts (15%) and SDW (85%). Across all other formulations, the proportions of plant extracts and SDW varied, while the proportion of the remaining ingredients—glacial acetic acid (2%), citric acid (1%), sodium chloride (3%), sodium bicarbonate (2%), sodium benzoate (0.5%), potassium sorbate (0.2%), lactic acid (3%), thyme essential oil (TEO) (0.2%), and nisin (0.1%)—were kept constant.

### 2.2. Estimation of Antimicrobial Compounds in Crude Plant Extracts

The available literature has demonstrated that the major antimicrobial compounds present in the jamun leaf extracts are polyphenols and tannins [19,38,39]. Therefore, in the present experiment, the concentration of both compounds was measured by following the method of Amorim et al. [40] using the Folin–Ciocalteu reagent (FCR) (S D Fine-Chem Limited, Vadodara, India)), a calibration curve (prepared with various concentrations of tannic acid, varying from 0.5 to 14.0 μg/mL, Figure S2) and UV–vis spectrophotometric readings at 760 nm by using a Beckman DU-640 spectrophotometer (Backman Coulter, Inc., Brea, CA, USA). The concentrations of both total polyphenols and total tannins were expressed in mg tannic acid equivalent/g of dry extract (mg TAE g^−1^ extract).

### 2.3. Selection of Base Material for Preparing Decontamination Wipes

To design the decontamination wipe (DW), 4 types of base sterile materials, namely, non-woven cloth, filter paper, muslin cloth, and tissue paper, were evaluated.

The final base material was selected based on the ease of application and the leftover residue of the base material on the meat surface after its use. It was confirmed that the selected material should not possess any inherent antimicrobial property. To check whether the selected material has any antimicrobial effects, it was tested against *S. aureus* and *E. coli* bacteria. The selected DS and the base material were used to prepare the final form of the DW. For that, a method was standardized and is presented in Figure S3.

### 2.4. Evaluation of Microbial Quality of Market Chicken Samples and Comparative Antimicrobial Effect of Developed Wipe Against Traditional Decontaminants

Retail chicken meat samples (N = 36) were collected randomly from the shops in Bareilly city, which include both halal and jhatka processed samples. By following the standard processes and maintaining hygiene, the samples were collected and carried to the laboratory in a refrigerated icebox. In the laboratory, the meat samples were processed for microbial count estimation (total plate count) as per standard procedure.

From the collected samples, 6 samples were taken out separately and divided into three groups (2 samples in each group): Group 1, meat treated with a wipe dipped in SDW (C1); Group 2, meat treated with a wipe dipped in 0.2% (*v*/*v*) sodium hypochlorite solution in distilled water (C2); and Group 3, meat treated with the developed DW (T). Before application of the wipe, swab samples were collected, and TPC was enumerated by following the standard process. For the treatment process, each wipe (C1, C2, and T) was rubbed separately over a 1 cm^2^ area of meat surface for 30 s prior to the swab sample collection. After applying the wipes, we waited for 2 min and then all the samples were processed for total plate count, calculating the log reduction due to the application of the DW. Although this short duration may not be sufficient for achieving the maximum antimicrobial effects of a biocidal agent, which typically requires a long contact period, it was intentionally chosen to simulate the practical retail handling conditions where wipes are basically used for rapid cleaning of surfaces rather than prolonged exposure.

### 2.5. Evaluation of the Effects of the DW on the Quality Attributes of Chicken Meat Samples

The wipe was applied on the chicken meat surface immediately after reaching the samples in the laboratory by following the standardized process (Figure S4). The effect of the DW was assessed by evaluating the changes in quality attributes during its refrigerated (4 ± 1 °C) storage under aerobic packaging conditions. A control chicken meat sample was also kept in a similar way but without being treated with the DW.

During the refrigerated storage, both samples were evaluated for their changes in various quality parameters, such as physicochemical, microbiological, and sensory quality. For the collection of samples, the schedule prescribed in Table 2 was followed.

#### 2.5.1. Evaluation of Quality Parameters of Stored Chicken Meat

Chicken samples were stored at 4 ± 1 °C, and at regular intervals of 1 day, the meat quality parameters were also assessed for both treatment and control.

##### pH

For pH determination, 5 g of meat was blended with 45 mL of distilled water for 1 min using an Ultra Turrax Tissue Homogenizer (Model T-25, IKA-Werke GmbH & Co. KG, Staufen im Breisgau, Germany). The homogenate developed by this process was used to measure its pH by dipping the glass electrode of the pH meter (Model CP 901, Century Instruments Ltd., Chandigarh, India) in it [41].

##### Extract Release Volume

To determine the extract release volume (ERV), 25 g of minced chicken was homogenized with 100 mL of distilled water using a tissue homogenizer (Ultra Turrax IKA, Model T18 Basic, IKA Wares Inc., Wilmington, NC, USA). The resultant slurry was filtered through a glass funnel lined with a Whatman filter paper No. 1. The filtrate was collected in the beaker, and the volume obtained in the first 15 min was measured as the ERV of the meat sample [42].

##### Total Volatile Basic Nitrogen

Total volatile basic nitrogen (TVBN) was estimated following the Conway diffusion method [43]. Briefly, 50 g of meat was homogenized with 2.5 g of trichloroacetic acid powder, and the slurry was filtered through a combined muslin cloth and Whatman^®^ filter paper No. 40. In the central compartment of a Conway microdiffusion unit, 2 mL of boric acid was taken, and in the outer compartment of this unit, 1 mL of meat filtrate was added. Further, 1 mL of saturated potassium carbonate solution was poured into the outer compartment, and the lid was closed. Then the whole system was incubated for 3–4 h at 37 °C. At the completion of the incubation period, the 0.02 N sulfuric acid was used for the titration of the boric acid solution. The formula, TVBN (mg/100 g) = (Vol. of 0.02 N sulfuric acid consumed × Normality of acid used for titration × 14 × 100), was used to assess the TVBN concentration.

##### Microbiological Parameters

For the microbial count of the meat surface, sterile swab sticks were used to apply over a 1 cm^2^ area every time. This surface area provides a small, fixed, and reproducible unit of measurement that allows microbial count to be expressed in CFU/cm^2^, which is a well-accepted reporting unit for surface contamination in meat microbiological studies [44].

Microbial counts of meat samples, including total plate count (TPC), psychrophilic count, Pseudomonas count, and yeast–mold count, were enumerated by following the APHA [45] method, and the results were expressed as log_10_ CFU/g. For sample preparation, 10 g of meat was blended with 90 mL of sterile 0.1% peptone water and homogenized for 15 min using an Ultra Turrax IKA (Model T18 Basic, IKA Wares Inc., Washington, DC, USA) tissue homogenizer to obtain a 10^−1^ dilution. Further, 1 mL of 10^−1^ dilution was taken to prepare 10^−2^ dilution by adding it to 9 mL of 0.1% peptone water. In a similar way, further dilutions were prepared by using 0.1% peptone water.

TPC was determined by using plate count agar. Plates were inoculated with 1 mL of aliquots from the serial dilutions and incubated at 37 ± 1 °C for 48 h. Plates yielding 30–300 colonies were counted. The obtained colony numbers were multiplied by the dilution factor and expressed as log_10_ CFU/g. By following the same process, plates were prepared for the psychrophilic count; only the incubation of the plates was performed at 4 ± 1 °C for 14 days. For yeast–mold count, meat samples were prepared similarly, and potato dextrose agar plates were used for culturing the microbes. After inoculation of 1 mL inoculum, the plates were incubated at 25 °C for 5–7 days. The colonies exhibiting black, white, yellow, and greenish-black color pigmentation were counted and presented as log_10_ CFU/g. To assess Pseudomonas count, Pseudomonas agar plates were inoculated with appropriately diluted inoculum and incubated at 25 ± 1 °C for 48 h, and plates with 30–300 colonies were counted, and after multiplying with the reciprocal dilution factor, they were presented as log_10_ CFU/g.

The swab samples were immersed in 9 mL sterile normal saline and vortexed for 30 s to uniformly disperse the microbes. Serial dilutions were prepared according to the method described by ICMSF [46], using sterile maintenance medium (0.9% NaCl and 0.1% peptone) up to 10^−6^ dilution. For total plate count, 10 µL aliquots from each dilution were spread onto the solidified plate count agar plate. Following incubation, colonies were counted, multiplied by the appropriate dilution factor, and expressed as log_10_ CFU/cm^2^.

A microbial spiking test, combined with the application of the DW on the chicken meat surfaces, aims to evaluate the effectiveness of the wipe in reducing microbial contamination. In the spiking test, chicken meat surfaces were inoculated with a known concentration (~10^7^–10^8^ CFU/mL) of *S. aureus* (ATCC 29213) and *E. coli* (ATCC 43888). After allowing sufficient time for microbial attachment, the DW was applied to the inoculated surfaces. The effectiveness of the DW treatment was assessed by comparing microbial reduction in treated samples with that of the control group (spiked with bacteria but not wiped with the DW). The process of the spiking test is presented in Figure S5.

##### Sensory Parameters

To evaluate the sensorial quality of the chicken meat samples, a sensory evaluation was conducted by a semi-trained sensory panel [47]. Different sensory attributes of chicken samples, including color, odor, texture, and OAA (overall acceptability), were evaluated 3 times by using the five-point descriptive scales. The sensory scores were leveled for all the attributes as follows: for color, 1 = extremely undesirable color to 5 = excellent color; for odor, 1 = extremely undesirable odor to 5 = excellent odor; for texture, 1 = extremely undesirable texture to 5 = excellent texture; and for overall acceptability, 1 = extremely undesirable to 5 = excellent [48].The sensory panel was constituted with 10 semi-trained panelists of different age groups of males and females from the scientists, masters, and PhD students of various divisions of IVRI from the University in Izatnagar, following the methods described by Meilgaard et al. [49]. At the time of sensory evaluation, meat samples were coded with three-digit numbers. Evaluation was conducted around 11:00–11:30 AM consistently. For this study, meat samples were procured from the Bareilly local market and used to assess their quality and further improvement. All panel members voluntarily participated in the sensory evaluation study and were informed about the objectives of the study and related facts. All procedures followed in this study were in full compliance with our institutional guidelines, although our institution does not mandate any ethics committee approval for the sensory evaluation of meat and meat products.

### 2.6. Statistical Analysis

Statistical analysis of the data obtained by quality evaluation of the meat samples was performed. All the experiments were repeated thrice, and data was analyzed using one-way ANOVA and presented as mean ± S.E. For that, statistical methods were applied by using SPSS^®^ software (V-17.0, Windows, SPSS Inc., Chicago, IL, USA) at a 95% significance level, whereas for all statistical analyses, the significant differences were calculated by using Duncan’s method [50] at *p* < 0.05 levels.

## 3. Results and Discussion

### 3.1. Antimicrobial Efficacy of the Selected Ingredients for Decontamination Solution

The sterilized deionized water (SDW) has a pH of about 5.4 [51], which is considered neutral to slightly acidic and is suitable in food systems, especially for washing food, without posing any significant risk to food safety or quality. Therefore, the use of SDW as the base component was found justifiable. The second most important component of the DS was jamun leaf extract. Previous studies have demonstrated many antimicrobial phytochemicals in jamun leaf extracts. Observations made by Shafi et al. [52] described that the jamun leaves can effectively cure many bacterial and fungal infections in humans. They showed that Jamun leaves’ essential oils could inhibit *E. coli*, *S. aureus*, *Pseudomonas*, and *Salmonella* effectively. Jagetia et al. [22] reported that the potential antimicrobial activity of aqueous Jamun leaf extracts may be attributed to the presence of bioactive compounds like phenolics, flavonoids, and tannins present in them. The jamun leaves contain gallic acid, ellagic acid, and many other polyphenols [38]. de Oliveira et al. [39] demonstrated a potent antimicrobial effect of polyphenols and tanninspresent in jamun leaf extracts against *E. coli* and a multi-resistant strain of *S. aureus*, with a MIC of 200 μmg/mL for both. Additionally, this leaf extract has been identified as effective against *Candida krusei*. Elfadil et al. [17] showed aqueous Jamun leaf extract as a potential broad-spectrum antimicrobial agent.

In the present experiment, the crude jamun leaf extracts contained major antimicrobial compounds, polyphenols, and tannins at 45.46 mg TAE g^−1^ dry extract and 44.97 mg TAE g^−1^ dry extract, respectively. Indriaty et al. [53] reported that the total tannin content in plant extracts varied between 8.17 mg TAE/g and 813.35 mg TAE/g (*p* < 0.05) and showed moderate to strong antimicrobial effects. Similarly, the phenolic fraction of jamun leaves has shown antimicrobial activity at higher concentrations (20 μg/mL) against the Gram-negative bacterium *Pseudomonas aeruginosa* and the Gram-positive bacterium *S. aureus* [54]. These observations support that the jamun leaf extracts, which contain a significant concentration of antimicrobial compounds, polyphenols, and tannins, could have a potent antimicrobial effect against the targeted microbes *S. aureus* and *E. coli*.

**Figure 1:**
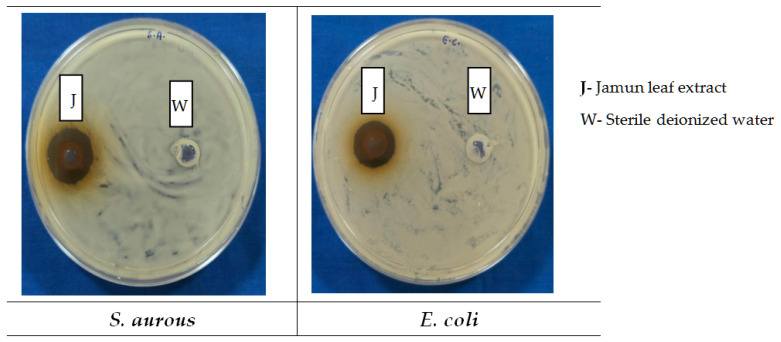
Well diffusion test for jamun leaf extracts against *S. aurous* and *E. coli*.

In this study, we used dried Jamun leaf powder for water extraction, which makes it safe to apply in the human food system as compared to any other extraction medium like alcohol [55]. Water extracts can be used directly in food without any concerns about negative health effects, while alcoholic extracts might need to be further processed or diluted for safe consumption [56]. Aqueous jamun leaf extracts exhibited a clear zone of inhibition measuring 5.5 mm against *S. aureus* and 4.0 mm against *E. coli* (Figure 1). The SDW as a negative control did not show any microbial inhibition zone for both of the tested organisms.

Studies have demonstrated that most antimicrobial medicinal plants show better antimicrobial efficacy against Gram-positive bacteria as compared to others [57]. The relatively complex cell structure of the Gram-negative bacteria may limit the penetration of the antimicrobial active compounds present in extracts, whereas the more permeable cell wall of Gram-positive bacteria facilitates higher susceptibility, resulting in more inhibition by extracts [58].

Organic acids have been approved as generally recognized as safe (GRAS) as food additives by the European Commission, FDA, and FAO/WHO [59]. Their antimicrobial effect is primarily attributed to their ability to lower pH and disrupt bacterial cell membranes. Mani-López et al. [24] showed that organic acids cause the flow of undissociated cellular components through microbial cell membranes. To cope with these changes, microbes try to maintain the intracellular pH, and hydrogen ions are released. The released ions lower the intracellular pH, leading to damage of enzymes, proteins, and DNA structure, as well as disruption of extracellular membranes, ultimately resulting in microbial cell death. The antimicrobial activity of sodium chloride is found to be due to its indirect effects on microbes by reducing water activity (a_w_) [60]. Sodium bicarbonate shows antimicrobial activity due to the alkaline pH created by the bicarbonate ions, which disrupts the cell membrane of bacteria and inhibits bacterial growth [61]. Sodium benzoate shows enhanced antimicrobial effects against pathogens like *E. coli*, *Salmonella*, and *Listeria* in lower pH [62]. This compound converts to benzoic acid in acidic environments, disrupts microbial cell membranes, and in hibitscellular metabolism. Sodium bicarbonate, a food-grade natural antimicrobial agent, has been found to be effective against *E. coli*, *Lactobacillus plantarum*, *S. aureus*, and *Pseudomonas aeruginosa* [63]. Sodium benzoate and potassium sorbate are widely used as food preservatives due to their proven antimicrobial activity [64]. Sorbate shows broad antimicrobial activity by inhibiting bacterial growth through mechanisms such as alteration of cell membranes, disruption of transport systems, inhibition of key enzymes, and introduction of proton flux into the cell [65]. The thyme EO contains the principal antimicrobial component thymol, which disrupts the bacterial cell membrane integrity, leading to leakage of intracellular contents and death of the bacterial cell [66,67]. Anzlovar et al. [68] reported strong antibacterial and antifungal activity of thyme essential oil, including effective inhibition of the growth of *E. coli*. Furthermore, thyme EO has shown promising antimicrobial activity against the microbes frequently isolated from food products, particularly poultry meat. Nisin is an important food-grade natural preservative, with strong antibacterial activity and minimal toxicity to humans. It is a ribosomally synthesized peptide with broad-spectrum efficacy against food-borne pathogens and is therefore widely applied as a food preservative throughout the world [69]. This natural preservative is effective against Gram-positive bacteria, with a minimum inhibitory concentration at nanomolar levels. Two primary mechanisms have been proposed for the antimicrobial action of nisin: pore formation in the cell membrane and inhibition of cell wall biosynthesis through binding to lipid II [70].

### 3.2. Formulation of DS and Evaluation of Its Antimicrobial Efficacy

**Figure 2:**
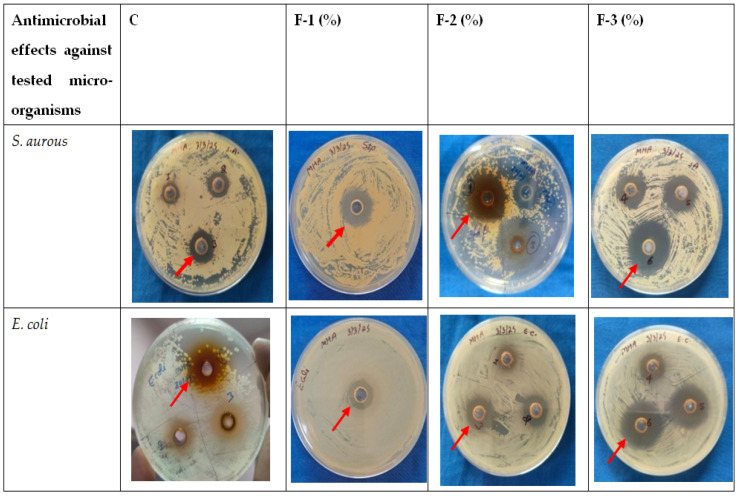
Antimicrobial effects of different DS formulations against tested microorganisms.

The antimicrobial efficacy of the developed DS was evaluated against test organisms *S. aureus* and *E. coli*, and corresponding zones of inhibition are presented in Figure 2.

Among the four DS formulations, the control (C; 85% SDW and 15% crude leaf extract) produced an inhibition zone of 3 mm against *S. aureus* and 2 mm against *E. coli*. Formulation F-1 (78% SDW and 10% crude leaf extract) produced a 5 mm zone of inhibition against *S. aureus* and 2 mm against *E. coli*. Formulation F-2 (73% SDW and 15% crude leaf extract) showed an 8 mm inhibition zone against *S. aureus* and 4 mm against *E. coli*. Formulation F-3 (68% SDW and 20% crude leaf extract) produced a 10 mm zone against *S. aureus* and 8 mm against *E. coli*.

The inhibition zone produced by the F-2 formulation was larger than that of the control and F-1. In comparison, F-3 produced the largest inhibition zones against both tested organisms. However, despite its better microbial inhibition properties, F-3 was not chosen, as it imparted an objectionable odor to the DS-treated chicken meat. Therefore, considering the antimicrobial efficacy and sensory acceptability, the F-2 formulation was chosen as the most suitable DS formulation.

### 3.3. Base Material for Decontamination Wipes (DWs)

**Figure 3:**
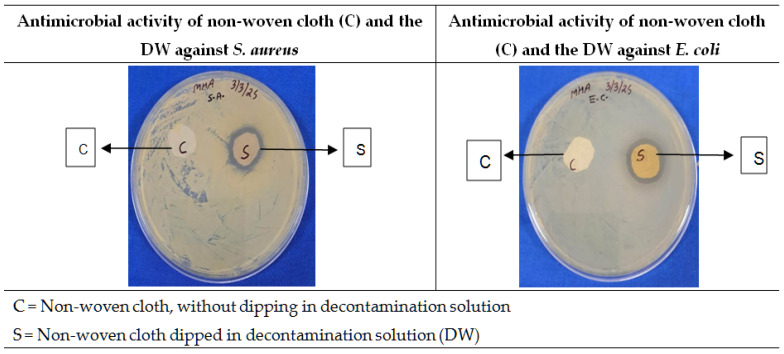
Assessment of antimicrobial effects of non-woven cloth and the developed DW.

Among the tested base materials for applying the DS, the non-woven cloth was found to be optimum based on the ease of application and neutrality, as it does not carry any antimicrobial effects and does not leave any residualfibers (critical in microbiologically sensitive environments) after application on the chicken meat surface. In addition, the non-woven cloth has a porous structure that allows it to absorb and uniformly retain liquid, ensuring effective delivery of the decontamination solution. This type of fabric maintains tensile strength even when saturated, preventing tearing during wiping. Excellent capillary absorption of liquid by the non-woven cloth and its subsequent release during wiping directly influence the efficacy of antimicrobial action. This material’s ability to absorb liquids effectively is a key characteristic used in various applications like hygiene products and filtration systems [71]. Das et al. [72] also reported extensive applications of the non-wovens as liquid absorbent materials, such as in diapers, sanitary napkins, etc. The neutrality of the non-woven cloth base with respect to antimicrobial activity is depicted in Figure 3.

The results showed no zone of inhibition against the tested microbes for ‘C,’ whereas ‘S’ showed a well-defined zone of inhibition. Masuku et al. [73] demonstrated that the cleaning efficiency of non-woven wipes on food-contact surfaces can be significantly enhanced when they are impregnated with the disinfectant solution. Here in our study, the developed DS showed excellent microbial-decontamination effects, and when it was soaked in the non-woven cloth wipe, it effectively increased the efficiency of the DW synergistically.

### 3.4. Assessment of the Microbial Quality of Market Chicken Meat Sample

**Figure 4:**
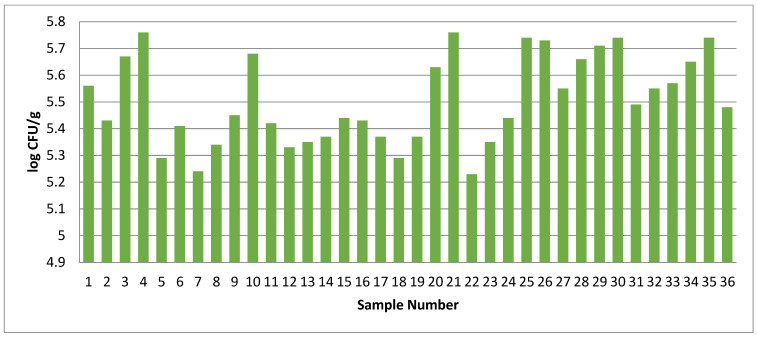
Total plate count (TPC, log CFU/g) of market chicken meat samples (N = 36).

Nutrient-rich chicken meat is highly prone to microbial contamination, and this process starts from the point of slaughter itself. The cleanliness and hygiene in retail are very crucial for the final quality of the meat [4]. In the present study, the total plate count of chicken samples was undertaken for all 36 chicken meat samples collected from the market. All the chicken meat samples were found to be highly contaminated, and the counts varied from 5.23 to 5.76 log_10_ CFU/g (Figure 4) but were under the acceptable limit as prescribed by FSSAI. Our finding also aligns with the findings of Huang et al. [74]; they reported TPCs between 3 and 6 log_10_ CFU/g for raw poultry meat samples.

### 3.5. Decontamination Effects of Developed DW

Prior to application, the wipes were taken out of the refrigerator (4 ± 1 °C) and conditioned at ambient temperature for 10 min before use. Although the thyme essential oil contains volatile compounds, its stability and antimicrobial efficacy did not become affected because of this environmental exposure, which may be due to its reported stability under moderate temperature [75]. Other than that, the oil was mixed with SDW and crude plant extracts and impregnated onto the non-woven cloth pieces; these substantially reduce the volatility of the compounds.

On day 1, when the chicken samples were collected, immediately upon reaching the laboratory, TPC was assessed before and after treating them with wipes (developed DW—T, wipe dipped in sterile deionized water—C1, and wipe dipped in 0.2% (*v*/*v*) sodium hypochlorite solution in distilled water—C2).After treatment with the respective wipes, a contact time of 2 min was allowed, followed by a second swab collection. Previous studies have demonstrated that a contact time of ≥5 min is generally required for optimal sanitizer efficacy; however, short contact wiping time has been shown to mechanically remove a substantial amount of surface microbiota [76]. Thus, this 2 min post-wiping contact time represents a compromise between practical applicability and experimental reproducibility.

The results presented in Table 3 showed a highly significant (*p* value = 0.00005, detection limit of the method being approximately 1 log CFU, CI-95%, 1.85, 2.77) log reduction (2.31) in microbial load after wiping the meat surface with the developed DW (count before treatment—5.48 log CFU/cm^2^; after treatment—3.17 CFU/cm^2^). On the other hand, the log reduction was comparatively lower (2.28 log, from an initial 5.52 log CFU/cm^2^; *p* value = 0.00007, detection limit of ~1 log CFU) in the sodium hypochlorite solution-treated wipe (C2) than the developed DW (T), although the values were close (*p* > 0.05). Meanwhile, the sterile deionized water (C1)-treated wipe could not reduce the load significantly (*p* > 0.05) after the treatment (*p*-value 0.87883).

### 3.6. Effect of Wipe Treatment on Quality Parameters of Chicken Meat During Storage at 4 ± 1 °C

Results showed a significant (*p* < 0.05) deterioration in the quality attributes of the stored chicken during storage at 4 ± 1 °C, and the data is presented in Table 4.

#### 3.6.1. Changes in Physicochemical Quality Parameters

A decrease (*p* < 0.05) in the pH of stored chicken meat was observed in both control (before intervention) and treatment (after intervention) samples. At the beginning of storage, the pH of chicken meat was recorded as 6.43 and 6.20, respectively. The initial pH declined gradually with increasing storage intervals, reaching an ultimate pH of 5.86 on day 5 for the control and 5.83 on day 6 for the treated chicken samples. The declining pH was comparable (*p* > 0.05) among storage days up to day 3 in control and up to day 5 in treatment samples. A significant (*p* < 0.05) decline was noticed from day 4 onwards in the control and from day 5 onwards in the treated sample. The treated samples showed significantly different (*p* < 0.05) pH values compared to the control on all the storage days except day 4. At the same time, there were visible signs of spoilage in both samples (day 5 in controls, day 6 in treatment). It has been observed that the time taken to reach the pH associated with spoilage signs was 5 days for the control and 6 days for the treatment. Meanwhile, treated meat samples also exhibited a significantly lower pH value than the control samples from day 1 to day 3 of storage. The acceptable pH limit for poultry meat, as suggested by Ristic and Damme [77], is pH 5.9 to 6.2. The gradually decreased pH in meat might be due to the myofibrillar and sarcoplasmic protein denaturation, resulting in increased contractions of actomyosin, which affected the meat structure [78]. A higher initial microbial load in meat due to unhygienic slaughtering and processing may cause accelerated meat spoilage and a faster rate of changes in meat pH [79]. Lactic acid bacteria were found responsible for the pH reduction in packaged fish [80]. It has also been observed that postmortem glycolysis of meat results in the lowering of pH during its long-term storage. Enzyme-induced myofibrillar structure degradation at the time of conditioning of meat might be responsible for the higher initial pH in the stored meat [81]. The lower pH values of treated meat samples might be due to the wiping of the meat with the DW, which contains organic acids like citric acid, lactic acid, and acetic acid, found responsible for lowering pH. It has been observed that organic acids release hydrogen ions (H^+^) when dissolved in water, which are responsible for the acidic nature of a solution. The same trend of observation has been quoted by Gonzalez-Fandos et al. [82], who studied the combined effects of organic acids and MAP on the *L. monocytogenes* in chicken legs during their storage at 4 °C.

In stored chicken, the TVBN concentrations (mg/100 g) increased significantly (*p* < 0.05) both in treated and control samples. The TVBN values were comparable (*p* > 0.05) up to day 2 of storage in both control and treated samples. After that, the TVBN content was found to be comparable (*p* > 0.05) among storage days from day 4 onwards in the control and from day 5 onwards in the treated samples. The treated samples showed significantly lower (*p* < 0.05) TVBN content as compared to the control, only up to day 2. The TVB-N has been treated as a potential biomarker of microbe-induced protein degradation in chicken meat [83]. As per the findings of Byun et al. [84], 20 mg/100 g of TVBN has been found as the acceptable limit in meat. Several other researchers have shown that the TVBN threshold used for spoilage indication is 20 mg/100 g [48,85]. In control and treatment chicken samples, the TVBN content gradually increased from its initial value of 8.64 and 7.76 mg/100 g, respectively, crossing the acceptable limit on day 5 and day 6 of storage, respectively, indicating the unacceptability of chicken meat. Upon further progress of the storage, the TVBN concentration further increased in both samples. The microbial deterioration of chicken causes the production of various volatile basic compounds like ammonia and other volatile basic gases due to the breakdown of the protein that is responsible for the increasing concentration of TVBN [48]. Lower TVBN concentrations in treated meat might be due to the antimicrobial property of the DW, and that is why the acceptable limit of TVBN was crossed later in the treated samples as compared to the control. Burfoot and Mulvey [86] showed a reduction (*p* < 0.05) in microbial count on the chicken and turkey carcasses using lactic acid wash and suggested improved shelf life and food safety. Findings of Soni et al. [87] showed the appearance of spoilage signs in refrigerated stored chicken on day 7, and the TVBN content reached 20.91 mg/100 g from its day 1 concentration of 7.64 mg/100 g.

Extract release volume (ERV) of meat is the amount of aqueous filtrate released from meat slurry after a fixed time. This measurement is used to assess the microbial spoilage of refrigerated fresh meat [88]. In the present study, during the storage of meat, the ERV decreased significantly (*p* < 0.05) in both control and treated chicken samples. The ERV values were found to be comparable (*p* > 0.05) among storage days from day 4 onwards in both meat samples. The treated samples showed significantly lower (*p* < 0.05) ERV values as compared to the control up to day 3 only. According to Miller [89], meat freshness can be directly assessed by its ERV value, which decreases with increasing storage intervals as a result of microbial degradation. According to Pearson [43], 17 mL is the acceptable limit of ERV for meat spoilage; therefore, it is a useful parameter to know the shelf life of meat. Better-quality meat with a lower microbial load shows more ERV than lower-quality meat [90]. Our findings revealed that the ERV value dropped below the reported acceptable limit (17 mL) on day 5 in the control and on day 6 in the treated samples. The proteolysis of meat protein due to microbial reasons during its storage might be the cause of decreased ERV [91]. The crossing of the acceptable ERV limit in the treated samples could be attributed to the antimicrobial effects of the developed DW, which effectively delayed microbial spoilage in this group. In addition, the chicken samples showed signs of spoilage on day 5 in control samples and on day 6 in treated samples. The findings of Selvan and Mendiratta [92] showed a higher value of ERV in lactic acid-treated buffalo liver as compared to the untreated liver. Our findings showed the acceptability of chicken meat when ERV was >17 mL, and that clearly reflected the antimicrobial efficacy of the DW.

#### 3.6.2. Changes in Microbial Quality Parameters

Different microbial quality attributes, like total plate count (TPC), psychrophilic count, *Pseudomonas* count, and yeast–mold count (log_10_ CFU/g), increased significantly (*p* < 0.05) during refrigerated storage of meat in both control and treated samples. The microbial spoilage and the subsequent freshness of meat may be influenced by multiple factors, including meat types, hygiene maintained at the time of slaughter, processing and handling, and storage temperatures. Among the various factors, the initial microbial load is considered the most important determinant influencing bacterial growth and spoilage in meat [93]. Correspondingly, visually detectable changes in meat quality were observed with the increase in microbial counts during storage.

In the control and treatment chicken samples, the initial TPC was 5.48 and 3.17 log_10_ CFU/g, respectively. The TPCs were comparable (*p* > 0.05) among storage days up to day 5 in control and up to day 2 in treated meat samples. In the control, a significantly (*p* < 0.05) higher count was noticed on day 5, whereas in treated samples, the count during days 3 to 5 increased gradually (*p* > 0.05), but a significant increase (*p* < 0.05) was noticed only on day 6. The treated samples consistently showed a significantly lower (*p* < 0.05) TPC than the control during storage days. The significantly lower (*p* < 0.05) microbial count (approximately 2 log reductions on day 1) observed in the treated samples compared to the control could be attributed to the application of the DW, which exerted antimicrobial effects. Researchers have shown the decontamination efficacy of organic acids [94], nisin [95], and thyme essential oil [96] on the chicken meat surface. With the increasing storage intervals, the TPC gradually rose to 6.04 on day 5 and 6.08 on day 6 in control and treated samples, respectively, coinciding with the appearance of spoilage signs in chicken meat. Simultaneously, the TPC reached beyond the acceptable limit suggested by FSSAI [97]. El Barbri et al. [98] also reported that meat spoilage occurs when the TPC exceeds 6 log_10_ CFU/g. The existing microflora proliferated on the chicken meat surface during storage and spoiled the control on day 5, but treatment on day 6. Since the initial microbial load is a major factor influencing chicken meat spoilage, with higher initial loads accelerating spoilage, this may explain why the treated samples reached spoilage one day later than the untreated (C) samples. As the fresh chicken meat has a limited shelf life under refrigerated conditions, microbial spoilage is of great concern for this [99]. Findings of Zhang et al. [100] showed an increment of total viable count (TVC) from 4.60 log_10_ CFU/cm^2^ to 5.38 and 6.38 log_10_ CFU/cm^2^ in chicken that was stored at 0–4 °C and 4 °C, respectively.

In this study, we observed a 1-day shelf-life extension, and though this improvement is quite small, it is still very important in the retail and food safety aspects. Fresh meat is a highly perishable food commodity, and therefore, a 1-day delay in its spoilage can reduce the risk of consumers’ exposure to high microbial loads and foodborne pathogenic microbes. In addition to that, a 1-day shelf-life extension can facilitate better distribution and marketing of fresh chicken meat and reduce the food waste, and subsequently, the economic loss can be avoided. Therefore, this shelf-life extension can add extra safety and quality benefits in chicken meat retailing.

During storage, the psychrophilic count in the chicken meat increased (*p* < 0.05) both in control and treated samples. The initial counts were 5.17 log_10_ CFU/g and 3.48 log_10_ CFU/g in the control and treated samples, respectively. The lower (*p* < 0.05) count in treated samples than the control might be due to the antimicrobial effects of the DW. The psychrophiles increased gradually (*p* > 0.05) from day 2 to day 5 in the control and up to day 5 in the treated meat samples. In treated samples, the count was significantly (*p* < 0.05) higher on day 6. The treated samples consistently showed a significantly lower (*p* < 0.05) count than the control up to day 5. With the increasing storage intervals, the psychrophiles gradually increased to 6.02 and 6.03 on day 5 and day 6 in the control and treatment, respectively. Up to this point of storage, the chicken meat had acceptable quality features and was sensorially appealing. After this point, the chicken began to exhibit visible signs of spoilage. Although there is no universally acceptable limit for psychrophilic count for chilled chicken meat, Mol et al. [101] suggested 6 log_10_ CFU/g as an acceptable limit for cold-stored foods. Based on this reference, the psychrophilic count exceeded the reported acceptable limit on day 5 in the control and on day 6 in the treated samples, indicating the antimicrobial effects of the DW.

The *Pseudomonas* count increased (*p* < 0.05) in the control and treated chicken samples during storage. Initial counts of 3.56 and 2.81 log_10_ CFU/g were observed in control and treatment samples, respectively. A lower (*p* < 0.05) count in the treated samples as compared to the control might be due to the antimicrobial effects of the DW. Different studies have also shown the levels of *Pseudomonas* spp. for chicken to be 2.7–3.8 [102]. During the storage period, the *Pseudomonas* count reached 5.45 and 5.50 log_10_ CFU/g in the control and treatment, respectively, at which point the chicken exhibited visually detectable spoilage signs. Being a psychrophilic bacterium, *Pseudomonas* multiplies more rapidly at lower temperatures (0–20 °C), as was observed in the present study. Findings of Raab and Kreyenschmidt [103] showed that for fresh aerobically stored poultry, *Pseudomonas* is a specific spoilage organism. Nychas et al. [104] showed *Pseudomonas* spp. as the dominant bacteria in aerobically stored meat between 0 and 25 °C temperatures.

An increment (*p* < 0.05) in yeast–mold count was also observed both in control and treatment chicken meat. The initial counts of 2.26 and 1.40 log_10_ CFU/g increased to 2.91 and 2.97 log_10_ CFU/g by day 5 and day 6 in control and treated samples, respectively, and at this point, the meat exhibited visually perceivable spoilage signs. In this study, the significantly lower (*p* < 0.05) count of yeast–mold in the treated samples as compared to the control might be attributed to the antimicrobial effects of the DW. An organoleptically rejected meat showed a yeast–mold count between log 5 and log 6 CFU/g [105]. Our findings were quitea bit lower than the referred threshold value, which indicates an insignificant contribution of yeast–mold in the spoilage of chicken meat in this experiment.

##### Evaluation of Antimicrobial Efficacy of DW by Spiking Test

**Figure 5:**
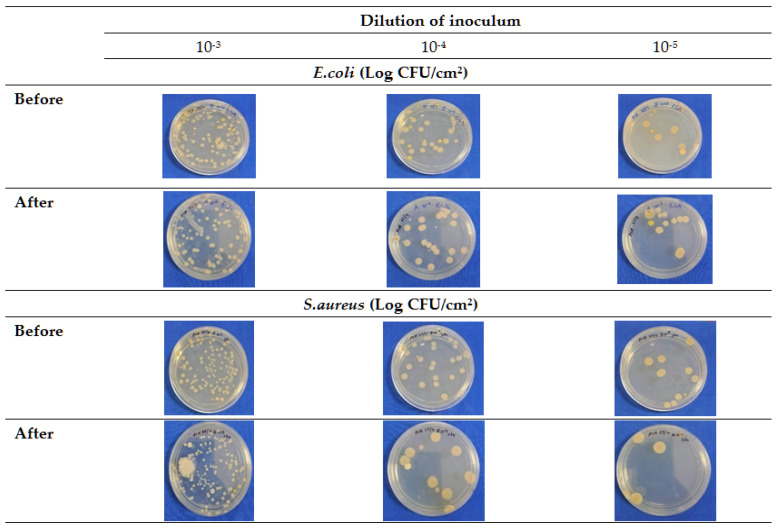
Reduction in microbial load (*E. coli*, *S. aureus*) detected by spiking test at various dilutions.

The outcome of the spiking test shows (Figure 5 and Table 5) that there was a significant reduction (*p* < 0.05) in both the *E. coli* (1.03 at 10^−5^ dilution, with *p*-00000, CI-95%, 0.90, 1.16) and *S. aureus* (1.14 at 10^−5^ dilution, with *p*-00000, CI-95%, 1.01, 1.25) counts across all the dilutions (10^−3^, 10^−4^, and 10^−5^). Reductions in microbial loads were >1 log_10_ CFU/cm^2^ in all dilutions, except at the 10^−4^ dilution for *S. aureus*.

In this study, the meat surface decontamination resulted in a higher log reduction in *E. coli* as compared to *S. aureus* with the application of the developed DW (Table 5). At the same time, a larger zone of inhibition (5.5 mm) was observed, made by the crude plant extract against *S. aureus*, as compared to the zone against *E. coli* (4.0 mm) via the well diffusion test (Figure 1). The well diffusion test assesses the diffusion of crude plant extracts through gel, which is not comparable to the meat surface log reductions achieved by using a multi-component decontamination wipe on the meat surface. Therefore, despite a large zone of inhibition by crude plant extracts (against *S. aureus*), the multi-component DW produced higher log reduction (1.14) for *E. coli* as compared to *S. aureus* (1.03), which reflects the synergy and matrix effects. Tayel et al. [106] demonstrated that a combination of various plant extracts can exhibit synergistic effects to show antimicrobial efficacy, resulting in more effective decontamination as compared to the effects of individual plant extracts.

#### 3.6.3. Sensory Quality Parameters

Tura et al. [107] showed that the sensory attributes of poultry products are important in deciding consumers’ choices. The sensory attribute scores for color, odor, texture, and overall acceptability for the stored chicken meat decreased significantly (*p* < 0.05) with the increasing storage intervals in both control and treated chicken samples. Initially, the chicken meat showed ‘characteristic grayish-pink to dull-red color’, with higher color scores (4.77 in control and 4.84 in treatment). Afterwards, a gradual decline in (*p* < 0.05) scores was noticed from day 3 onwards in both samples. Score declined (*p* < 0.05) further on day 5 and day 6 to 0.91 and 0.95 in control and treated samples, respectively. At this stage, the color score changed from ‘excellent’ to ‘extremely undesirable,’ coinciding with the appearance of visible spoilage in chickens. Across the storage intervals, significantly higher (*p* < 0.05) color scores in treated samples compared to control may be attributed to the antimicrobial effects of the DW, which may have reduced microbial degradation and maintained better meat color in treated samples. Notably, when the color score was above 2.00, the TPC was under the acceptable limit. A score below 1 corresponded to the TPC exceeding the acceptable limit. Microbe-induced change in oxymyoglobin to brown-colored metmyoglobin might have resulted in a lower color score. In addition to this, surface drying of chicken meat at the time of storage might have also contributed to lower color scores [108]. It has been observed that the metabolites produced by the existing microbes in the meat resulted in physicochemical changes in the meat, contributed to the discoloration and unpleasant smell, and formed mucus [109]. These unacceptable sensory changes were observed less in treatment chicken samples, which might be due to the antimicrobial effects of the DW.

For the odor score, a significant (*p* < 0.05) reduction was observed both in control and treated samples, with the lowest scores (0.83 and 0.87, respectively) corresponding to the visible spoilage signs in chicken. The score declined (*p* < 0.05) on day 3 onwards in the control and on day 4 onwards in the treated samples. Finally, the scores reduced (*p* < 0.05) to around 0.91 and 0.95 on days5 and 6 of storage in control and treated meat samples, respectively. This score depicts the unacceptability of chicken, which was manifested with an off-odor. The off-odor in chicken might be due to the lipid oxidation and microbial metabolites produced due to protein degradation [110]. The lower odor (*p* < 0.05) scores observed in the control samples compared to the treatment samples may be attributed to the absence of the DW application, which allowed higher microbial degradation of the meat components. This microbial activity resulted in the production of off-odor metabolites, lowering odor scores. Consistent with the findings of Ayres et al. [111], psychotropic bacteria are responsible for producing off-odor compounds (sulfurous compounds) in spoiled poultry, which might have contributed to the lower odor score and unacceptability of the control samples.

The texture scores of the stored chicken meat samples were found to decrease significantly (*p* < 0.05) with the increasing storage intervals in both control and treated chicken samples. Higher initial scores (4.58 for control and 4.84 treatment) declined (*p* < 0.05) on day 3 of storage in control and on day 4 in treated samples. Finally, the texture score decreased to 0.62 and 0.54 in control and treated samples on days 5 and 6, respectively, indicating a change in scores from “excellent texture” to “extremely undesirable texture.” The findings in this study indicated that the storage time significantly hampers meat texture, which was visible in raw chicken meat. The declining texture score may be due to the structural changes in proteins caused by the decreasing pH and protein degradation [112]. This might be induced by the microbes existing in the meat. A significantly higher (*p* < 0.05) texture score in treated samples compared to control may be attributed to the DW, which might have reduced microbial degradation of the meat and helped in preserving a relatively intact texture. During cold storage conditions, weakening of the muscle structure occurs due to changes in the post-rigor mortis phase, affecting meat texture. Consistent with the findings of Erkmen and Faruk Bozoglu [113], maintaining optimal storage conditions can partially prevent the degradation of texture quality.

The overall acceptability scores of chicken meat reduced significantly (*p* < 0.05) from their initial higher values, 4.66 and 4.83 for control and treatment to 0.83 and 0.54 on days 5 and 6, respectively. Decreased scores for color, odor, and texture resulted in lower overall acceptability scores. Our findings showed an unacceptable overall acceptability score, which coincided with the appearance of visible spoilage signs in chicken and TPC beyond the acceptable limit.

## 4. Conclusions

Unhygienic retail practices contribute significant microbial contamination to chicken meat surfaces. This meat surface contamination further adversely affects the overall quality of the meat and shortens the shelf life of the products. An easy-to-use, safe, economical, and environmentally friendly meat surface decontamination process may help both retailers and consumers to assure safety and prevent economic losses. Therefore, a decontamination solution was prepared using a blend of SDW, crude jamun leaf extracts, thyme essential oil, nisin, and other GRAS ingredients in appropriate proportions. This solution was soaked in a non-woven cloth to prepare the meat surface DW for convenient application over the meat surface. The application of the DW was found to reduce 2.31 log of the microbial load (TPC) over the chicken surface, as compared to the non-treated meat. During the storage of the meat in aerobic packaging at refrigeration temperature, the treated meat was found to have improved physicochemical, microbiological, and sensory quality as compared to the non-treated meat. Further, the treated meat had a 1-day extended shelf life as compared to the non-treated meat. This 1-day shelf life extension can reduce the risk of consumers’ exposure to high microbial loads and foodborne pathogenic microbes. In addition, this can facilitate a better distribution and marketing of fresh chicken meat and reduce the food waste, and subsequently, the economic loss. The spiking test demonstrated a significant decontamination efficacy of the DW against pathogens *S. aureus* and *E. coli*. Therefore, the developed DW proved to be a very effective way to decontaminate the retail chicken meat surface and extend the shelf life of the fresh chicken meat during its refrigerated storage without compromising the sensory attributes.

## Figures and Tables

**Table 1:** Formulations of decontamination solution (DS).

Sl. No.	Ingredients	C	F-1 (%)	F-2 (%)	F-3 (%)
1.	Sterilized deionized water (SDW)	85	78	73	68
2.	Plant extracts	15	10	15	20
3.	Glacial acetic acids	-	2	2	2
4.	Citric acid	-	1	1	1
5.	Sodium chloride	-	3	3	3
6.	Sodium bicarbonate	-	2	2	2
7.	Sodium benzoate	-	0.5	0.5	0.5
8.	Potassium sorbate	-	0.2	0.2	0.2
9.	Lactic acid	-	3	3	3
10.	Thyme essential oil (TEO)	-	0.2	0.2	0.2
11.	Nisin	-	0.1	0.1	0.1
Total		100	100	100	100

**Table 2:** Evaluation of quality change in chicken meat stored at refrigeration temperature (4 ± 1 C°).

Storage Temperature	Intervals of Study	Storage Period						
		Day 1	Day 2	Day 3	Day 4	Day 5	Day 6	Until Spoilage
4 ± 1 °C	1 day regular interval	Physicochemical, microbiological, and sensory quality attributes						

Note: The DW was applied to the treatment meat samples on day 1, while control samples were not treated with the wipe. Both groups were then stored at 4 ± 1 °C, in aerobic packaging separately.

**Table 3:** Changes in total plate count after application of various wipes over meat surface.

TPC (log CFU/cm^2^)	C1		C2		T	
B	5.53 ^A^ ± 0.11	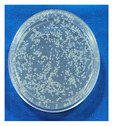	5.52 ^A^ ± 0.12	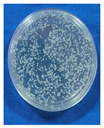	5.48 ^A^ ± 0.14	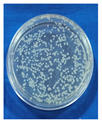
A	5.52 ^A^ ± 0.11	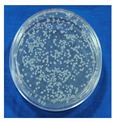	3.24 ^B^ ± 0.25	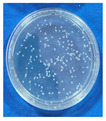	3.17 ^B^ ± 0.25	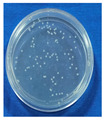
LR	0.01 log		2.28 log		2.31 log	
*p*-value	0.87883		0.00007		0.00005	
CI	95%, −0.17, 0.19		95%, 1.79, 2.76		95%, 1.85, 2.77	

n = 6; (mean ± SE) with different superscripts column-wise (upper case) indicate significant (*p* < 0.05) difference. C1—wipe dipped in sterile deionized water, C2—wipe dipped in 0.2% (*v*/*v*) sodium hypochlorite solution in distilled water, T—developed DW, B—before application of wipe, A—after application of wipe, LR—log reduction, and CI—confidence interval.

**Table 4:** Changes in the chicken meat quality parameters during storage at refrigeration temperature (4 ± 1° C).

Quality Attributes		Storage Period						F-Value
		Day 1	Day 2	Day 3	Day 4	Day 5	Day 6	
Physicochemical Parameters								
pH	B	6.43 ^aA^ ± 0.03	6.40 ^aA^ ± 0.01	6.30 ^abA^ ± 0.07	6.03 ^bcA^ ± 0.08	5.86 ^cA^ ± 0.09	5.75 ^cA^ ± 0.12	13.45
	A	6.20 ^aB^ ± 0.03	6.18 ^aB^ ± 0.03	6.14 ^aB^ ± 0.02	6.07 ^abA^ ± 0.06	6.00 ^abB^ ± 0.07	5.83 ^bA^ ± 0.08	5.378
Total Volatile Basic Nitrogen (mg/100 g)	B	8.64 ^dA^ ± 0.19	10.44 ^cdA^ ± 0.86	13.78 ^bcA^ ± 1.70	17.51 ^abA^ ± 0.90	20.06 ^aA^ ± 0.88	20.33 ^aA^ ± 0.53	26.44
	A	7.76 ^dB^ ± 0.19	9.61 ^dB^ ± 0.85	13.30 ^cA^ ± 1.06	17.06 ^bA^ ± 0.50	19.58 ^abA^ ± 0.66	20.04 ^aA^ ± 0.28	60.00
Extract Release Volume (mL)	B	23.69 ^aA^ ± 0.69	22.51 ^aA^ ± 0.33	19.67 ^bA^ ± 0.15	17.62 ^cA^ ± 0.37	16.83 ^cA^ ± 0.32	15.94 ^cA^ ± 0.59	49.12
	A	21.95 ^aB^ ± 0.73	21.16 ^aB^ ± 0.72	20.05 ^abB^ ± 0.23	18.49 ^bcA^ ± 0.26	17.22 ^cA^ ± 0.39	16.77 ^cA^ ± 0.47	17.08
Microbiological Parameters (log CFU/g)								
Total Plate Count (TPC)	B	5.48 ^bA^ ± 0.13	5.51 ^bA^ ± 0.33	5.63 ^abA^ ± 0.28	5.91 ^abA^ ± 0.27	6.04 ^abA^ ± 0.11	6.74 ^aA^ ± 0.32	3.34
	A	3.17 ^eB^ ± 0.25	3.69 ^deB^ ± 0.10	4.13 ^cdB^ ± 0.03	4.72 ^bcB^ ± 0.23	5.35 ^abB^ ± 0.26	6.08 ^aB^ ± 0.28	25.16
Psychrophilic Count	B	5.17 ^bA^ ± 0.07	5.28 ^abA^ ± 0.25	5.63 ^abA^ ± 0.14	5.87 ^abA^ ± 0.26	6.02 ^abA^ ± 0.22	6.14 ^aA^ ± 0.25	3.37
	A	3.48 ^cB^ ± 0.43	3.72 ^bcB^ ± 0.36	3.91 ^bcB^ ± 0.51	4.56 ^abcB^ ± 0.55	5.3 ^abB^ ± 0.15	6.03 ^aA^ ± 0.25	6.07
*Pseudomonas* Count	B	3.56 ^cA^ ± 0.16	4.09 ^bcA^ ± 0.39	4.76 ^abcA^ ± 0.17	4.94 ^abA^ ± 0.15	5.45 ^aA^ ± 0.28	5.63 ^aA^ ± 0.15	7.49
	A	2.81 ^eB^ ± 0.23	3.05 ^deB^ ± 0.33	3.81 ^cdB^ ± 0.14	4.17 ^bcB^ ± 0.22	4.94 ^abA^ ± 0.19	5.50 ^aA^ ± 0.20	20.86
Yeast and Mold Count (YMC)	B	2.26 ^aA^ ± 0.67	2.40 ^aA^ ± 0.80	2.48 ^aA^ ± 1.19	2.59 ^aA^ ± 0.85	2.91 ^aA^ ± 1.59	3.08 ^aA^ ± 1.26	2.13
	A	1.40 ^bB^ ± 0.67	1.70 ^abB^ ± 0.80	2.08 ^abA^ ± 0.95	2.30 ^abA^ ± 1.50	2.53 ^abA^ ± 0.54	2.97 ^aA^ ± 0.86	2.88
Sensory Evaluation								
Color	B	4.77 ^aA^ ± 0.06	4.41 ^aA^ ± 0.12	3.68 ^bA^ ± 0.17	2.25 ^cA^ ± 0.09	0.91 ^dA^ ± 0.19	0.87 ^dA^ ± 0.12	160.90
	A	4.84 ^aB^ ± 0.08	4.52 ^aB^ ± 0.20	3.70 ^bA^ ± 0.08	2.73 ^cB^ ± 0.09	2.16 ^cB^ ± 0.24	0.95 ^dB^ ± 0.16	85.50
Odor	B	4.62 ^aA^ ± 0.15	4.25 ^abA^ ± 0.11	3.66 ^bA^ ± 0.35	2.62 ^cA^ ± 0.12	0.83 ^dA^ ± 0.10	0.66 ^dA^ ± 0.15	81.82
	A	4.95 ^aB^ ± 0.04	4.29 ^abA^ ± 0.10	3.75 ^bA^ ± 0.28	2.70 ^cA^ ± 0.13	1.29 ^dB^ ± 0.16	0.87 ^dA^ ± 0.10	107.20
Texture	B	4.58 ^aA^ ± 0.15	4.33 ^aA^ ± 0.16	3.66 ^bA^ ± 0.16	2.58 ^cA^ ± 0.20	0.62 ^dA^ ± 0.12	0.41 ^dA^ ± 0.05	145.20
	A	4.84 ^aB^ ± 0.10	4.41 ^abA^ ± 0.15	3.75 ^bB^ ± 0.11	2.83 ^cB^ ± 0.27	1.08 ^dB^ ± 0.15	0.54 ^dA^ ± 0.10	119.20
Overall Acceptability	B	4.66 ^aA^ ± 0.21	4.37 ^aA^ ± 0.15	3.66 ^bA^ ± 0.16	2.50 ^cA^ ± 0.18	0.83 ^dA^ ± 0.16	0.29 ^dA^ ± 0.04	127.70
	A	4.83 ^aB^ ± 0.10	4.50 ^abB^ ± 0.18	4.08 ^bB^ ± 0.08	2.91 ^cB^ ± 0.15	1.1.6 ^dB^ ± 0.24	0.54 ^dB^ ± 0.15	122.50

n = 6; df = 5, 25; (mean ± SE) with different superscripts row-wise (lower case) and column-wise (upper case) indicate significant (*p* < 0.05) difference; B = before application of wipe; A = after application of wipe.

**Table 5:** Effects of the DW on the meat surface microbial load detected by the spiking test.

	Dilution of Inoculum		
	10^−3^	10^−4^	10^−5^
*E. coli* (Log CFU/cm^2^)			
Before	6.09 ^A^ ± 0.00	6.32 ^A^ ± 0.01	6.88 ^A^ ± 0.02
After	5.02 ^B^ ± 0.01	5.26 ^B^ ± 0.04	5.74 ^B^ ± 0.04
Log reduction	1.06 ± 0.00	1.06 ± 0.03	1.14 ± 0.02
*p*–value	0.00000	0.00000	0.00000
CI	95%, 1.02, 1.10	95%, 0.94, 1.18	95%, 1.01, 1.25
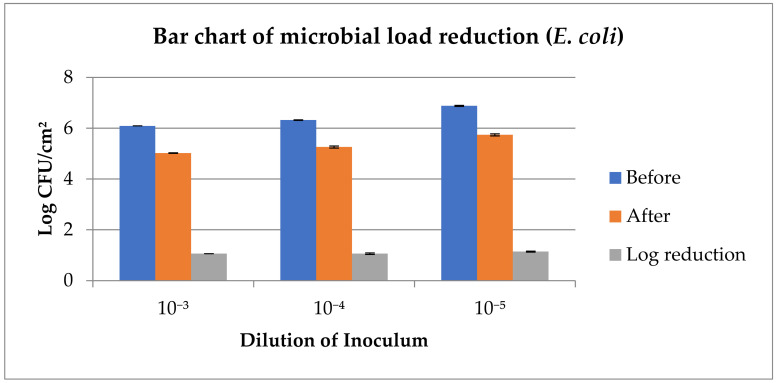			
***S. aureus* (Log CFU/cm^2^)**			
Before	6.08 ^A^ ± 0.01	6.45 ^A^ ± 0.25	7.07 ^A^ ± 0.03
After	5.06 ^B^ ± 0.03	5.65 ^B^ ± 0.20	6.03 ^B^ ± 0.02
Log reduction	1.01 ± 0.02	0.80 ± 0.04	1.03 ± 0.00
*p*-value	0.00000	0.01451	0.00000
CI	95%, 0.93, 1.09	95%, 0.24, 1.36	95%, 0.90, 1.16
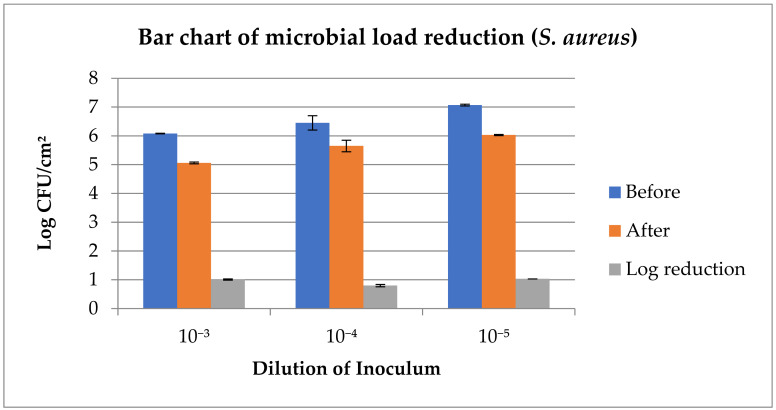			

n = 6; (mean ± SE) with different superscripts column-wise indicates significant (*p* < 0.05) difference; B = before intervention; A = after intervention.

## Data Availability

Data is available from the corresponding author on reasonable request.

## Supplementary Materials

The supporting information can be downloaded at https://www.mdpi.com/article/10.3390/foods14193391/s1.
