# Supplementary material for: Enhancing Chicken Meat Quality with User-Friendly Decontamination Wipes
Source: Foods. 2025 Sep 30;14(19):3391. doi: 10.3390/foods14193391 (PMC12523915; doi:10.3390/foods14193391)
Supplement: Supplementary file 1 [file foods-14-03391-s001.zip › foods-3843019-supplementary.pdf]

Supplementary materials

Plant parts were collected and clean with running tap water

⇩

Were gently patted dry with tissue paper and put in the preheated hot air oven at 37°C for 6 hours

⇩

The dried parts were blended with mixture and grinder into fine powder

⇩

Powdered plant parts were added with deionized water to make up concentration of 40% each in 250 mL conical flask and covered with aluminium foil to avoid exposure to direct light and evaporation

⇩

After thorough mixing, the mixture was kept un disturbed for 1 day at room temperature

⇩

The mixture in conical flask were put in shaking water bath at 55°C at 70 rpm, for 24 hours

⇩

Then the mixture was transferred to a 50 mL centrifuge tube and centrifuge for 10 min at 6000 rpm

⇩

The supernatant was collected in covered conical flask and kept at 4° C for further use

Figure S1. Preparation of water extracts from jamun leaves.

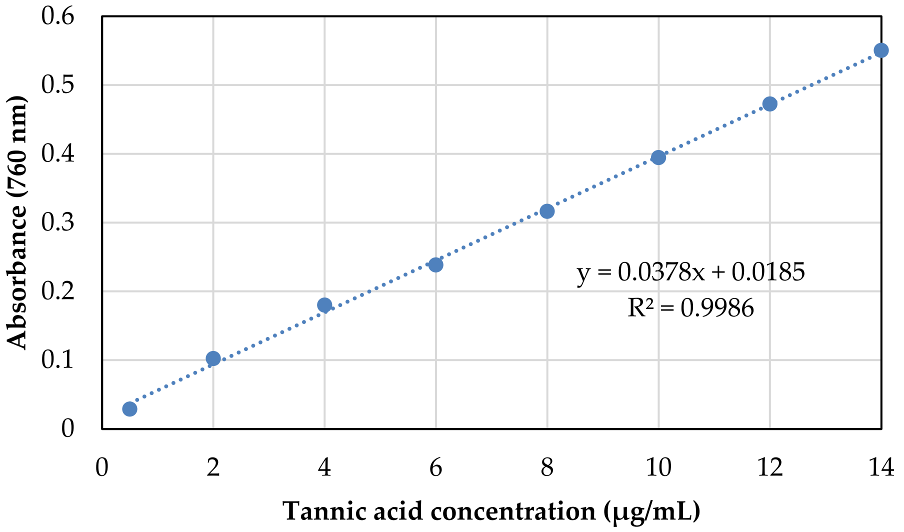

Figure S2. Standard curve for estimation of antimicrobial compounds in crude plant extracts.

Base materiel was cut into round shapes (1 cm diameter)

⇩

Made them sterile by autoclaving

⇩

Soak the base materiel in the DS by dipping in it, with the help of a sterile forceps

⇩

Kept the base in the DS for 10 sec

⇩

Stake 10 no. of the base material soaked in DS on above another in a sterile container

⇩

Pour 10 mL decontamination solution in the container

⇩

After wrapping in the aluminium foil the container kept in refrigerator (4±1° C) for further use

Figure S3. Preparation of decontamination wipes.

Chicken meat samples purchase from retail shops and brought to the laboratory in ice box by maintaining all precautionary measures

⇩

Immediacy after reaching to the laboratory test was conducted

⇩

The DW was brought out from refrigerator and kept in ambient temperature for 10 min for conditioning before its use

⇩

With help of sterile swab, on the meat piece surface swabbing was made with the DW

⇩

One DW was took out of the stake and wiped all over the surface of the chicken meat piece thoroughly, with the use of hand gloves

⇩

Kept the meat piece undisturbed for 2 min

⇩

With sterile swab, meat piece surface swabbing was made in an area of 1 cm^2

⇩

Total plate count was estimated for both control and treatment

⇩

Wiped chicken meat samples (treatment) and the non-wiped meat samples (control) were packaged aerobically in LDPE pouches and stored in refrigerator for further study

Figure S4. Evaluation the efficacy of DW.

Fresh culture of *E. coli* and *S. aureus* was grown in Brain Heart Infusion (PBI) broth for 18-24 h

⇩

Centrifuge the culture at 8000 rpm for 10 min to make a pellet

⇩

Pellet was washed two times with Phosphate Buffered Saline (PBS) and centrifuged at 8000 rpm for 10 min

⇩

Resuspended the pellet in 30 mL PBS to achieve ~10^7–10^8 CFU/mL bacterial concentration using plate count confirmation

⇩

Two medium size (30 g) meat pieces (for control-C, and treatment -T) were taken

⇩

Both the meat pieces were dipped in boiling water for 2-3 sec to make them sterile

⇩

The surface of both pieces were treated with 1 mL bacteria culture and incubated for 45 min to allow bacterial adherence to the meat surface

⇩

The treatment meat surface (T) (1 cm^2 area) was wiped with DW and excess solution was drained and let the piece air dry briefly under sterile condition and swab samples were taken from outer surface of both C & T meat carefully

⇩

The meat pieces were put in separate stomacher bags and 225 mL PBS was added and stomach for 2 min

⇩

Serial dilutions (10^{-1} to 10^{-5})was maed and spread plates were prepared from each dilution in MacConkey agar for *E. coli*, and Mannitol Salt agar for *S. aureus* then incubated both at 37°C for 24 h

⇩

Colonies were counted using formulae to calculate CFU/cm^2

⇩

Log reduction = $\log_{10}$(CFU/g in control)−$\log_{10}$(CFU/g in treatment), was calculated

$$\textbf{CFU/cm}^2 = \frac{\text{No. of colonies x Total dilution factor}}{\text{Volume of culture plates in mL}}$$

Figure S5. Performance of spiking test.
